# Risk Factors for Development of Acute Kidney Injury in Critically Ill Patients: A Systematic Review and Meta-Analysis of Observational Studies

**DOI:** 10.1155/2012/691013

**Published:** 2012-11-26

**Authors:** Rodrigo Cartin-Ceba, Markos Kashiouris, Maria Plataki, Daryl J. Kor, Ognjen Gajic, Edward T. Casey

**Affiliations:** ^1^Division of Pulmonary and Critical Care Medicine, Mayo Clinic, 200 First Street SW, Rochester, MN 55905, USA; ^2^Department of Anesthesia, Mayo Clinic, 200 First Street SW, Rochester, MN 55905, USA; ^3^Division of Nephrology and Hypertension, Mayo Clinic, 200 First Street SW, Rochester, MN 55905, USA

## Abstract

*Background*. Acute kidney injury (AKI) is a frequent complication of critically ill patients. The impact of different risk factors associated with this entity in the ICU setting is unknown. *Objectives*. The purpose of this research was to assess the risk factors associated with the development of AKI in critically ill patients by meta-analyses of observational studies. *Data Extraction*. Two reviewers independently and in duplicate used a standardized form to collect data from published reports. Authors were contacted for missing data. The Newcastle-Ottawa scale assessed study quality. *Data Synthesis*. Data from 31 diverse studies that enrolled 504,535 critically ill individuals from a wide variety of ICUs were included. Separate random-effects meta-analyses demonstrated a significantly increased risk of AKI with older age, diabetes, hypertension, higher baseline creatinine, heart failure, sepsis/systemic inflammatory response syndrome, use of nephrotoxic drugs, higher severity of disease scores, use of vasopressors/inotropes, high risk surgery, emergency surgery, use of intra-aortic balloon pump, and longer time in cardiopulmonary bypass pump. *Conclusion*. The best available evidence suggests an association of AKI with 13 different risk factors in subjects admitted to the ICU. Predictive models for identification of high risk individuals for developing AKI in all types of ICU are required.

## 1. Introduction 

Acute kidney injury (AKI) is a common and highly lethal problem faced in the intensive care unit (ICU) [1, 2], with a reported incidence of 1 to 67% [3–6], and a mortality that ranges from 28 to 90% [5, 7–10]. This wide range in incidence and mortality is in part due to the near 35 different definitions of AKI [11]. Different risk factors for the development of AKI in the intensive care unit (ICU) have been assessed in diverse populations, including port-surgical, trauma, and medical patients. A wide variety of risk factors have been described but there is no clear understanding of what risk factors confer the highest risk for development of AKI. In addition, risk factors identified in some studies have not been confirmed in subsequent studies or the effect may differ depending on the clinical setting. Better understanding the impact and the association of different risk factors with AKI is of paramount importance for designing predictive models of high risk patients, and also to create preventive strategies that might benefit patients from developing this lethal condition. Predictive models for development of AKI already exist in cardiac-surgery critically ill patients [12–14]; however, refinements are still required to modify these studies into clinically applicable tools, and there is lack of meaningful predictive models in mixed and medical ICUs where most of the prediction models have focused on the impact on mortality of AKI in ICU patients [15, 16]. Given this situation, how can we identify patients at risk for AKI and are there interventions to mitigate this risk? To answer the former question in order to be able to respond the latter one, we set out to conduct a systematic review of the risk factors associated with the development of AKI in the ICU.

## 2. Methods

The report of this protocol-driven systematic review and meta-analysis adheres to the Preferred Reporting Items for Systematic Reviews and Meta-Analyses (PRISMA) standards for reporting systematic reviews and meta-analysis studies [17].

### 2.1. Study Eligibility

Eligible studies were observational studies (prospective and retrospective cohorts or case-control studies) where specific risk factors for AKI were investigated in critically ill adult (≥18) patients with no AKI at the time of ICU admission. Studies were required to have a control group with no AKI for comparison and the main outcome of interest was development of AKI by any definition during the ICU stay (definition must include criteria based on change in creatinine levels). Study inclusion was not limited by publication status or language. Exclusion criteria included studies assessing specific causes of AKI such as rapidly progressive glomerulonephritis, hemolytic-uremic syndrome, or hepatorenal syndrome, and also specialized ICUs (burn and transplant ICUs). Studies must have excluded end stage kidney disease patients.

### 2.2. Search Strategy

An expert medical librarian with extensive meta-analytical experience collaborated to design the search strategies. The following databases were searched since inception of the database until the third week of January of 2010: Ovid MEDLINE, Ovid EMBASE, Cochrane Library, Web of Science, and Scopus. Database-specific controlled language and terms that describe the key concepts were utilized: acute kidney injury, acute renal failure, acute renal insufficiency, risk factors, intensive care unit, critically ill, outcomes, and observational studies. The detailed search strategy is described in Appendix 1, in supplementary material available online at doi:10.1155/2012/691013. We also reviewed the reference sections of identified reviews, published guidelines, and published manuscripts known to the authors. In addition, we reviewed the reference sections of eligible studies.

### 2.3. Study Selection

Using a high threshold for exclusion, pairs of reviewers, working independently and in duplicate, identified potentially eligible studies for full-text retrieval from the titles and abstracts. Studies in which the reviewers disagreed were also retrieved in full text. Subsequently, disagreements were harmonized by consensus; when this wasnot possible, an independent reviewer reviewed and resolved the discordance by arbitration. We assessed interobserver agreement by the kappa statistics. Two independent native French speakers reviewed 2 studies published in French; similarly, 2 independent native Spanish speakers reviewed 2 studies published in Spanish.

### 2.4. Data Extraction

Two investigators independently used standardized forms to abstract descriptive, methodological, and outcome data from all eligible studies. The reviewers utilized a standardized data extraction form that include characteristics of the individual studies (sample size, gender distribution), clinical setting (surgical (trauma, cardiac, or general), mixed or medical ICU) and country, number of centers involved in the study, type of study (prospective or historical cohort, case-control study), years of enrollment, definition of AKI utilized, incidence of AKI in the study, and risk factors for AKI. Because of significant inconsistency in reporting risk factors among the studies, we assessed the following risk factors deemed to be of significant importance in the developing of AKI in all the eligible studies: age, diabetes, hypertension, heart failure, baseline serum creatinine (as a measure of evidence of chronic kidney disease), sepsis/systemic inflammatory response syndrome (SIRS), nephrotoxic drugs (including intravenous contrast dye, aminoglycosides, amphotericin B, vancomycin, nonsteroidal anti-inflammatory drugs, angiotensin converting enzyme inhibitors, and angiotensin receptor blockers), severity of disease as measured by different severity scores such as the acute physiologic and chronic health evaluation (APACHE) [18, 19] or the injury severity score (ISS) [20], hypotension or shock, use of vasopressors and/or inotropes, high risk surgery, and emergency surgery. High risk surgery and emergency surgery were not assessed in medical ICUs for obvious reasons. For cardiosurgical ICUs, we also assessed the use of intra-aortic balloon pump (IABP) and the time on the cardiopulmonary bypass pump in minutes.

### 2.5. Quality Assessment

Independent reviewers working in duplicate determined the quality of each study based on the Newcastle-Ottawa quality assessment scale (NOS) for cohorts and case control studies [21]. Disagreements were harmonized by consensus. When this was not possible, a third reviewer adjudicated the quality of the questionable study.

### 2.6. Statistical Analysis

The main outcome of assessment was the development of AKI. The risk estimate analyzed for every risk factor in the studies was the odds ratio (OR) for dichotomous variables, and the mean difference for the continuous variables with its corresponding 95% confidence interval (CI). When available, we used the adjusted risk estimates (adjusted OR) from multivariate models. For severity of disease, because of diverse severity of disease scoring systems, we estimated the point estimate for each study using the effect size (ES), which expresses the effect of the risk factor in terms of the standard deviation of the measurement producing a unitless estimate that can be compared across studies [22]. We performed separate meta-analyses with the random effects model [23] to obtain the pooled OR or pooled mean difference for the development of AKI for each risk factor with its corresponding 95% CI. We then used the *I* ^2^ statistic to quantify the proportion of observed inconsistency across study results not explained by chance [24]. *I* ^2^ values of less than 25%, 50%, and more than 75% represent low, moderate, and high inconsistency, respectively [24]. We proposed predefined subgroup analyses based on our a priori hypotheses to explain potential heterogeneity across studies on the strength of association due to different ICU types (surgical, mixed, and medical), considering a significant interaction when *P* < 0.05. In addition, in order to evaluate the impact of studies with poor methodological quality, we conducted sensitivity analyses excluding studies in the lower 2 quartiles of the NOS quality assessment scale scores. Comparisons of risk estimates between subgroups and sensitivity analyses were made with a test of interaction [25]. The presence of publication bias was investigated graphically by the method of Sterne and Egger [26]. All analyses were performed with Review Manager Software (RevMan Analyses Version 5.0.4 Copenhagen; The Nordic Cochrane Center, The Cochrane Collaboration, 2008).

## 3. Results

### 3.1. Search Results and Study Inclusion

**Figure 1:**
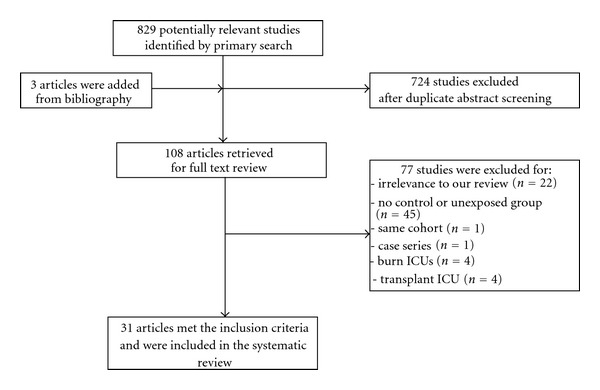
Flow diagram of the process of study selection.

Our initial search identified 829 unique publications in the form of titles and abstracts, which were narrowed by preliminary review to 108 potentially relevant original articles that were examined in full text. The interobserver agreement in this phase was *κ* = 0.90 (95% CI, 0.88–0.93). The search of references from the 108 retrieved papers identified 3 additional articles.  Figure 1 describes the flow diagram of the process of study selection and the reasons for exclusion of studies. Ultimately, our systematic review and meta-analysis included 31 studies [4–6, 27–54] that evaluated a total of 504,535 individuals.

Eleven studies were prospective cohorts, sixteen studies were historical cohorts, three studies were case-control studies, and one study was a nested case-control study. Most of these studies were done in surgical ICUs (*n* = 20; 14 of which were cardiothoracic ICUs); the remainder were mixed ICUs (*n* = 8), and medical ICUs (*n* = 3). There were a wide range of years of enrollment of the cohorts, from 1976 to as recent as 2008. A total of 26 different definitions of AKI were identified in these 31 studies, all had in common a prespecified elevation of creatinine or an elevation of creatinine from baseline. The incidence of AKI in the different ICUs as defined by the multiple definitions ranged from 1.2 to 67%.  Table 1 describes the studies' characteristics in detail. 

### 3.2. Methodological Quality

Table 2 summarizes the quality of the studies utilizing the NOS quality assessment scale. The median score of the studies was 6 (interquartile range, 6 to 8), where most of the studies failed to demonstrate that the outcome (AKI) was not present at start of the study. Moreover, only 14 studies performed a multivariate analysis for adjustment of baseline imbalances and confounders. 

### 3.3. Meta-Analyses

Separate meta-analyses for each risk factor demonstrated that all assessed risk factors, with the exception of hypertension, were significantly associated with the development of AKI in critically ill patients as depicted in Table 3. There was a trend of association of hypertension with AKI; however, it was not statistically significant (OR 1.15, 95% CI 0.76, 1.74). After excluding 4 studies that assessed hypertension due to poor quality (see sensitivity analyses), no interaction was observed but the risk estimate was significant (OR 1.43 95% CI 1.08, 1.89), and there was also an improvement in heterogeneity between studies. Higher levels of creatinine at baseline and a higher severity of disease score (equivalent difference of 18 points in the APACHE III score, 9 points in the ISS, and 6 points in the APACHE II score) were also associated with AKI. Besides these associations, patients in cardiothoracic ICUs that developed AKI showed a significant association with the use of IABP (OR 3.29, 95% CI 2.21, 4.91) and with longer time on the cardiopulmonary bypass pump (mean difference 27.92, 95% CI 14.41, 41.43 minutes). Table 3 also describes the reporting of the different risks appraised. It is noticeable that with the exemption of age, “reporting bias” is very suggestive in the remainder risk factors. Very few risk factors were also reported as part of multivariate adjustment and, when available, these results were included in the analyses. Significant heterogeneity existed between studies among the different risk factors evaluated (*I* ^2^ > 75%), with the only exemption of nephrotoxic drugs that presented moderate heterogeneity with an *I* ^2^ of 42%.

### 3.4. Subgroup Analyses

Preplanned subgroup analyses were performed for every risk factor according to the type of ICU (surgical, mixed, or medical).  Table 4 demonstrates that significant interaction was only observed in diabetes and heart failure between mixed ICUs, and high risk/emergency surgery in surgical and mixed ICUs. The point estimate association of diabetes and heart failure in the development of AKI in mixed ICUs remained significant but lower than the pooled estimate. Patient characteristics' differences regarding the inclusion of both medical and surgical patients in the mixed ICUs might account for the observed discrepancy, and less likely the studies included in the subgroup analyses which presented low heterogeneity for both diabetes (*I* ^2^ 0%) [34, 49, 50, 53] and heart failure (*I* ^2^ 0%) [4, 34, 50, 53]. Furthermore, outcomes of patients that develop AKI in surgical ICUs tend to be worse than in medical ICUs [9].

The effect of high risk/emergency surgery between surgical and mixed ICUs likely represents opposite effects of the two types of ICUs in the pooled point estimate. The estimate of association with AKI is not significant for high risk/emergency surgery in mixed ICUs as opposed to surgical ICUs, where the point estimate is even higher. A possible explanation could be that the risk is likely diluted due to the inclusion of both “surgical” and “medical” patients in mixed ICUs. To confirm this hypothesis, we excluded the study with higher reported number of medical patients as opposed to surgical patients in the mixed with ICUs [4] and the resulted OR was 1.52 (95% CI 1.10, 2.09), with a test for interaction that was not significant (*P* = 0.09).

### 3.5. Sensitivity Analyses

We performed sensitivity analyses to test how robust the results of the meta-analyses were in relation to the methodological quality of the studies. Studies with a NOS score <6 were excluded in our sensitivity analyses. Our results were not significantly altered by the exclusion of studies with poor methodological quality, with mild inconsistency improvement, particularly in diabetes, heart failure, nephrotoxic drugs, and cardiopulmonary bypass time as described in Table 5.

### 3.6. Publication Bias

The funnel plots for every individual risk factor are presented in Appendix 2, in supplementary material available online at doi:10.1155/2012/691013. Most of the plots showed asymmetry, suggesting small-study bias (either the absence of or inability to find studies with smaller or negative risk estimates) or unexplained heterogeneity. 

## 4. Discussion

This study found that the current evidence, drawn from a large number of observational studies that included more than half million individuals, indicates a significantly increased risk of AKI in critically ill patients with older age, diabetes, higher baseline creatinine, heart failure, sepsis/SIRS, use of nephrotoxic drugs, higher severity of disease scores, use of vasopressors/inotropes, high risk surgery, emergency surgery, and possibly hypertension. Additionally, cardiothoracic patients admitted to the ICU also presented increased risk of AKI with the use of IABP and longer time in cardiopulmonary bypass pump. We found that many of these observational studies were methodologically limited and presented high levels of heterogeneity. Multiple definitions of AKI, differences in populations studied, differences in the internal characteristics and practice of the diverse ICUs, differences in countries, and differences in processes of care over time might explain the inconsistencies identified in this review. Most of the definitions of AKI utilized in these studies possess high specificity for the diagnosis of AKI but poor sensitivity due to lack of urine output criteria [55]. With the exemptions already described, the risk factors described in this review that were associated with AKI presented similar point estimates across different types of ICUs. It was reassuring that after exclusion of methodological limited studies, all point estimates remained significant. Our findings demonstrated important methodological issues observed in several of the studies analyzed, particularly the potential impact of “reporting bias” and lack of adjustment for important covariates in observational studies. 

### 4.1. Limitations/Strengths

Our results are limited to the extent that the observational studies included in this systematic review yielded inconsistent and imprecise data about association effects. One might argue the legitimacy of combining dissimilar patients from different types of ICUs. Our analysis was subject to the limitation of different definitions of AKI among the studies (Table 1), which in fact may have contributed to the unexplained heterogeneity that we have identified between our selected studies. This differential approach in defining AKI could have potentially overestimated or underestimated some the risk factors identified in our analysis. However, the subgroup analysis looking at each type of ICU did not result in significantly different point estimates for the vast majority of the risk factors. Furthermore, in all systematic reviews, the usual key limitation is publication and reporting bias. Our review clearly showed evidence of significant reporting bias and possible publication bias. The strengths of this systematic review stem from the exhaustive literature search, a thorough evaluation of the methodological quality of the studies, and a focused analysis with complete, prespecified subgroup, and sensitivity analyses. Furthermore, the most compelling evidence comes from our pooled analysis of studies that included thousands of patients. Finally, our contact of the study authors to obtain missing data and their collaboration with our request further strengthen the quality of the review.

### 4.2. Clinical Implications and Future Research

Our findings should be considered for designing prediction models of AKI in different ICUs with the overarching goal of implementing strategies to prevent this highly lethal and morbid condition. The results provided in this review are also applicable to clinical practice and counseling of patients at high risk of development of AKI. From the research perspective, the potential role of diabetes and heart failure in critically ill AKI patients from different ICU types warrant further investigation. 

## 5. Conclusions

While meta-analyses of observational studies carry significant limitations inherent to the design of the studies, this review assessed and confirmed the association of 13 different risk factors in the development of AKI in critically ill adults from 31 studies of more than half million patients. This review demonstrated a significantly increased risk of AKI in critically ill patients with older age, diabetes, hypertension, higher baseline creatinine, heart failure, sepsis/SIRS, use of nephrotoxic drugs, higher severity of disease scores, use of vasopressors/inotropes, high risk surgery, emergency surgery, use of IABP, and longer time in cardiopulmonary bypass pump. Early identification of patients at risk of AKI may help to implement interventions to mitigate this highly lethal condition.

## Supplementary Material

Appendix 1 includes the detailed keywords and search strategy employed by the expert librarian in the following databases: Ovid MEDLINE, Ovid EMBASE, Cochrane Library, Web of Science, and Scopus.
Appendix 2 includes the funnel plots employed for critically appraising the publication bias for each identified risk factor for acute kidney injury with the respective subgroups, which include the ICU setting: surgical, medical or mixed.

## Figures and Tables

**Table 1:** Description of studies included in the systematic review and meta-analysis.

First author, year	Setting/country	Study design/number of centers	Years of enrollment	Total sample, % men	AKI definition	Incidence of AKI in the study	Severity of disease score, mean standard deviation (SD)
Hilberman 1979 [43]	Surgical-Cardiac/USA	PC/1	1977-1978	226/NR	Need for RRT of peritoneal dialysis or BUN > 70 mg/dL or GFR < 30 mL/min/m^2^	14.6%	NR
Wilkins and Faragher 1983 [53]	Mixed/UK	HC/1	1976–1979	475/51	Creatinine > 180 *μ*mol/L and BUN > 8 mmol/L for >24 h	23%	NR
Corwin 1989 [37]	Surgical-Cardiac/USA	CC/1	1985	84/NR	↓ Creatinine 50% of baseline	N/A	NR
Boucher 1990 [32]	Surgical-Trauma/USA	CC/1	1988-1989	184/76	↑ Creatinine ≥ 0.5 mg/dL from baseline or ↑ creatinine > 50% baseline	N/A	ISS (28 ± 13)
Groeneveld 1991 [42]	Medical/Netherlands	HC/1	1986-1987	487/52	↑ Creatinine ≥ 3.2 mg/dL or ↑ creatinine more than 2 times the baseline	16%	NR
Tran 1994 [51]	Surgical-Trauma/Netherlands	HC/1	1985–1989	206/73	↑ Creatinine ≥ 3.2 mg/dL or ↑ creatinine more than 2 times the baseline	15%	ISS (39 ± 17.6)
Zanardo 1994 [54]	Surgical-Cardiac/Italy	PC/1	1989–1999	734/68	↑ Creatinine ≥ 2.5 mg/dL	3.7%	NR
Ward 1996 [52]	Medical/USA	HC/1	NR	102/53	↑ Creatinine ≥ 0.5 mg/dL from baseline	17.6%	APACHE II (18.7 ± 1.8)
Vivino 1998 [5]	Surgical-Trauma/Italy	PC/1	1991–1994	153/80	↑ Creatinine ≥ 2 mg/dL or ↑ creatinine > 20% of baseline	31%	ISS (28.1 ± 11)
Mangano 1998 [48]	Surgical-Cardiac/USA	PC/24	1991–1993	2,222/76	↑ Creatinine > 177 *μ*mol/L or ↑ creatinine > 62 *μ*mol/L from baseline or need for RRT	7.7%	NR
Conlon 1999 [36]	Surgical-Cardiac/USA	PC/1	1995–1997	2,672/64	↑ Creatinine ≥ 1 mg/dL from baseline	8%	NR
De Mendonça 2000 [4]	Mixed/16 countries	PC/40	May 1995	1411/NR	↑ Creatinine ≥ 3.5 mg/dL and/or urine output < 500 mL/24 hours	24.7%	NR
Ficher 2002 [40]	Surgical-Cardiac/Germany	CC/1	1999-2000	143/73	↑ Creatinine ≥ 0.5 mg/dL from baseline or RRT	N/A	NR
Clermont 2002 [35]	Mixed/USA	PC/1	NR	1530/NR	↑ Creatinine ≥ 0.5 mg/dL if baseline ≤ 1.9 mg/dL, or ↑ creatinine ≥ 1 mg/dL if baseline ≥ 2 to ≤ 4.9 mg/dL, or ↑ creatinine ≥ 1.5 mg/dL if baseline ≥ 5 mg/dL	17.2%	APACHE III (64 ± 31.8)
Bove 2004 [33]	Surgical-Cardiac/Italy	PC/1	1998–2002	5,068/67	↑ Creatinine ≥ 100% from baseline	3.4%	NR
Bahar 2005 [30]	Surgical-Cardiac/Turkey	HC/1	1991–2001	14,437	↑ Creatinine ≥ 100% from baseline 24 hours after surgery or sustained urine output < 20 mL/hour	1.2%	NR
Chawla 2005 [34]	Mixed/USA	PC/1	2002-2003	194/53	↑ Creatinine > 75% from baseline if creatinine ≤ 2 mg/dL or ↑ creatinine > 50% from baseline if creatinine > 2 mg/dL	18%	APACHE II (12.5 ± 5.6)
Loef 2005 [46]	Surgical-Cardiac/Netherlands	HC/1	1991	843/73	↑ Creatinine ≥ 25% of baseline creatinine during first week after surgery	17.2%	NR
Mataloun 2006 [49]	Mixed, Brazil	PC/1	1999-2000	221/46	↑ Creatinine ≥ 1.5 mg/dL	19%	APACHE II (15.2 ± 7.7)
Hoste 2006 [6]	Mixed/USA	HC/1	2000-2001	5,383/51	RIFLE	67%	APACHE III Median (IQR) (56, 41–73)
Landoni 2007 [45]	Surgical-Cardiac/Italy	PC/1	1998–2003	3,103/84	↑ Creatinine > 100% from baseline	2.2%	NR
Barrantes 2008 [31]	Medical/USA	HC/1	2005	381/57	↑ Creatinine ≥ 0.3 mg/dL or ≥ 50% from baseline and/or an episode of < 0.5 mL/Kg/hr urine output for > 6 hours despite fluid challenge of ≥ 500 mL of normal saline	31.5%	APACHE II (15 ± 5.85)
Bagshaw 2008 [28]	Mixed/Australia and New Zealand	HC/57	2000–2005	120,123/59	RIFLE (only cases in first 24 hours of admission), modified urine output criteria	36%	APACHE II (16.9 ± 7.7)
Bagshaw 2008 [29]	Surgical-Trauma/Australia and New Zealand	HC/57	2000–2005	9,449/70	RIFLE (only cases in first 24 hours of admission), modified urine output criteria	18.1%	APACHE II (17.5 ± 7.6)
Dasta 2008 [38]	Surgical-Cardiothoracic/USA	NCC/1	1998–2002	3,741/63	RIFLE creatinine criterion	6.9%	APACHE III (54 ± 17.3)
Abelha 2009 [27]	Surgical-NonCardiac/Portugal	HC/1	2006–2008	1,166/65	↑ Creatinine ≥ 0.3 mg/dL or ≥ 50% from baseline and/or an episode of < 0.5 mL/Kg/hr urine output for >6 hours despite a ≥500 ml fluid challenge	7.5%	APACHE II median (IQR) (8, 5 to 11)
De Ara*ú*jo Brito 2009 [39]	Surgical-Cardiac/Brazil	HC/1	2003–2006	186/59	↑ Creatinine > 50% from baseline if creatinine > 1.3 mg/dL or ↑ creatinine > 0.5 mg/dL from baseline if creatinine < 1.3 mg/dL, or need of RRT	30.6%	NR
Thakar 2009 [50]	Mixed/USA	HC/191	2001–2006	325,395/97	↑ Creatinine ≥ 0.3 mg/dL from baseline	22%	NR
Hobson 2009 [44]	Surgical-Cardiothoracic/USA	HC/1	1992–2002	2973/66	RIFLE creatinine criterion	43%	NR
Machado 2009 [47]	Surgical-Cardiac/Brazil	HC/1	2003–2008	817/70	↑ Creatinine ≥ 0.3 mg/dL or ≥50% from baseline	48.5%	EuroSCORE, median (IQR) 1.8 (1.2–3)
Gomes 2010 [41]	Surgical-Trauma/Portugal	HC/1	2001–2007	436/80	RIFLE	50%	ISS (27.3 ± 11.4)

AKI: acute kidney injury, SD: standard deviation, BUN: blood urea nitrogen, GFR: glomerular filtration rate, HC: historical cohort, PC: prospective cohort, CC: case-control, NCC: nested case control, RRT: renal replacement therapy, NR: not reported, IQR: interquartile range, ISS: injury severity score, APACHE: acute physiologic and chronic health evaluation score, RIFLE: risk injury failure loss end stage kidney disease criteria, EuroSCORE: European System for Cardiac Operative Risk Evaluation.

To convert *μ*mol/dL to mg/dL, divide by 88.

**Table 2:** Quality of the studies utilizing the Newcastle-Ottawa quality assessment scale (maximum score of 9).

Cohort studies									
	Selection				Comparability	Outcome			Total score
First author, year	Representativeness of exposed cohort	Selection of the nonexposed cohort	Ascertainment of exposure	Demonstration that outcome was not present at start of study	Comparability on the basis of design or analysis	Assessment of outcome	Followup long enough	Adequacy of followup of cohorts
Hilberman 1979 [43]	∗		∗			∗	∗		4
Wilkins 1983 [53]	∗	∗	∗			∗	∗	∗	6
Groeneveld 1991 [42]	∗	∗	∗		∗	∗	∗	∗	7
Tran 1994 [51]	∗	∗	∗			∗	∗	∗	6
Zanardo 1994 [54]	∗	∗	∗	∗	∗	∗	∗	∗	8
Ward 1996 [52]			∗		∗	∗			3
Vivino 1998 [5]	∗	∗	∗	∗	∗	∗	∗	∗	8
Mangano 1998 [48]	∗	∗	∗	∗	∗	∗	∗	∗	8
Conlon 1999 [36]	∗	∗			∗	∗	∗	∗	6
De Mendonça 2000 [4]	∗	∗	∗		∗	∗	∗	∗	7
Clermont 2002 [35]	∗	∗	∗			∗	∗	∗	6
Bove 2004 [33]	∗	∗	∗	∗	∗	∗	∗	∗	8
Bahar 2005 [30]	∗	∗			∗	∗	∗	∗	6
Chawla 2005 [34]			∗	∗	∗	∗	∗	∗	6
Loef 2005 [46]	∗	∗	∗			∗	∗	∗	6
Mataloun 2006 [49]	∗	∗		∗	∗	∗	∗	∗	7
Hoste 2006 [6]	∗	∗	∗		∗	∗	∗	∗	7
Landoni 2007 [45]	∗	∗	∗		∗	∗	∗	∗	7
Barrantes 2008 [31]	∗	∗		∗		∗	∗	∗	6
Bagshaw 2008 [28]	∗	∗	∗			∗		∗	5
Bagshaw et al., 2008 [29]	∗	∗	∗			∗		∗	5
Abelha 2009 [27]			∗		∗	∗		∗	4
De Araújo Brito 2009 [39]			∗		∗	∗		∗	4
Thakar 2009 [50]		∗				∗	∗	∗	4
Hobson 2009 [44]	∗	∗	∗	∗		∗	∗	∗	7
Machado 2009 [47]		∗				∗	∗	∗	4
Gomes 2010 [41]	∗	∗	∗			∗	∗	∗	6

Case-control studies									
	Selection				Comparability	Exposure			Total score
First author, year	Case definition adequate	Representiveness of cases	Selection of controls	Definition of controls	Comparability on the basis of designs or analysis	Ascertainment of exposure	Same ascertainment method	Nonresponse rate

Corwin 1989 [37]	∗	∗			∗				3
Boucher 1990 [32]	∗	∗		∗	∗∗	∗	∗	∗	8
Ficher 2002 [40]	∗				∗	∗	∗		4
Dasta 2008 [38]	∗	∗			∗	∗	∗	∗	6

**Table 3:** Risk factors for acute kidney Injury evaluated in 31 observational studies.

	Age (years)	Diabetes	Hypertension	Baseline creatinine (mg/dL)	Heart failure	Sepsis/ SIRS	Nephrotoxic drugs	Severity of disease	Hypotension/ shock	Pressors/ inotropes	High risk surgery/ emergency surgery	Cardiopulmonary bypass time (minutes)	IABP
Hilberman 1979 [43]	*●*											*●*	
Wilkins 1983 [53]	⚪	⚪			⚪	*●*	*●*		*●*	*●*		N/A	N/A
Corwin 1989 [37]	*●*	⚪	⚪	*●*	*●*		⚪						⚪
Boucher 1990 [32]	⚪			⚪		⚪	*●*	⚪	*●*			N/A	N/A
Groeneveld 1991 [42]	⊙					*●*					N/A	N/A	N/A
Tran 1994 [51]	⚪					*●*		*●*				N/A	N/A
Zanardo 1994 [54]	⊙									*●*	⊙	*●*	⊙
Ward 1996 [52]	*●*			⊙				⊙			N/A	N/A	N/A
Vivino 1998 [5]	⊙					⚪	⚪	⊙	*●*			N/A	N/A
Mangano 1998 [48]	⊙	⊙^!^	*●*	⊙	⊙				⊙	*●*		⊙	*●*
Conlon 1999 [36]	⊙	⊙		⊙	*●*							⊙	*●*
De Mendonça 2000 [4]	⊙				⊙				⊙		⚪	N/A	N/A
Ficher et al., 2002 [40]	⚪	⚪	⚪							*●*			*●*
Clermont 2002 [35]	⚪							*●*	*●*			N/A	N/A
Bove 2004 [33]	⊙	*●*		⊙	⊙						⊙	⊙	⊙
Bahar 2005 [30]	⊙	*●*	*●*	*●*						*●*	*●*	⊙	⊙
Chawla 2005 [34]	⚪	⚪	⚪		⚪	⊙	⚪	*●*	⚪		⚪	N/A	N/A
Loef 2005 [46]	*●*	⚪	⚪	⚪	⚪		⚪				*●*	*●*	*●*
Mataloun 2006 [49]	⚪	⚪	⚪	⚪		*●*	⚪	⚪	⊙	*●*	⚪	N/A	N/A
Hoste 2006 [6]	⊙			⊙				⊙				N/A	N/A
Landoni 2007 [45]	⊙	⚪		⊙	⊙						⊙	*●*	*●*
Barrantes 2008 [31]	*●*				*●*	*●*	*●*	*●*			N/A	N/A	N/A
Bagshaw et al., 2008 [28]	*●*			*●*		*●*		*●*			*●*	N/A	N/A
Bagshaw 2008 [29]	*●*			*●*				*●*				N/A	N/A
Dasta 2008 [38]	⚪*	⚪		*●*	*●*			⚪*					
Abelha 2009 [27]	*●*		⚪		⊙			*●*			⊙	N/A	N/A
De Araújo Brito 2009 [39]	⚪		⚪	⚪					⚪	*●*		*●*	*●*
Thakar 2009 [50]	*●*	*●*	*●*	*●*	*●*							N/A	N/A
Hobson 2009 [44]	*●*	⚪	⚪	*●*	*●*							*●*	*●*
Machado 2009 [47]	*●*	*●*		*●*				*●*				⚪	⚪
Gomes 2010 [41]	⚪							*●*				N/A	N/A
Number of studies pooled for Meta-analysis	26	15	9	10	14	8	7	10	8	7	10	9	11
Number of patients	172,710	359,163	347,484	31,740	346,503	122,240	2,341	133,668	5,046	18,418	147,384	27,984	33,198
OR (95% CI)		1.52 [1.27, 1.82]	1.15 [0.76, 1.74]		1.97 [1.65, 2.35]	3.56 [1.85, 6.85]	1.54 [1.14, 2.08]		2.89 [1.54, 5.42]	4.75 [2.51, 8.99]	2.34 [1.59, 3.43]		3.29 [2.21, 4.91]
Mean difference (95% CI)	5.36 [3.72, 6.99]			0.22 [0.02, 0.41]				7.09 [4.86, 9.32]				27.92 [14.41, 41.43]	
*I* ^2^	97%	80%	96%	99%	74%	95%	42%	100%	84%	88%	94%	94%	80%

^*∗*^Cases and controls were matched on age and APACHE III. ^!^Only type 1 diabetes assessed.

SIRS: systemic inflammatory response syndrome, IABP: intra-aortic balloon pump, CI: confidence interval, OR: odds ratio.

⚪ Risk assessed, no significant difference between groups.

*●* Risk assessed, significant difference between groups in unadjusted comparison.

⊙ Risk assessed, significant difference between groups in adjusted comparison.

N/A: not applicable (risk assessed only for cardiac surgery studies).

**Table 4:** Subgroups analysis.

Risk Factor	Number of studies	Odds ratio or mean difference* (95% CI)	*P* value for interaction	Inconsistency *I* ^2^
Age				
Surgical	17	5.35 [2.84, 7.86]	0.99	96%
Mixed	6	4.95 [2.27–7.63]	0.81	97%
Medical	3	7.61 [4.77, 10.44]	0.17	0%
Diabetes				
Surgical	11	1.61 [1.41, 1.83]	0.61	31%
Mixed	4	1.09 [1.07, 1.11]	<0.001	0%
Hypertension				
Surgical	7	1.34 [1.04, 1.73]	0.53	68%
Mixed	2	0.68 [0.45, 1.05]	0.08	49%
Baseline creatinine*				
Surgical	8	0.20 [−0.02, 0.42]	0.89	99%
Heart Failure				
Surgical	9	2.19 [1.92, 2.5]	0.34	8%
Mixed	4	1.51 [1.47, 1.55]	0.003	0%
Sepsis/SIRS				
Surgical	2	2.69 [1.28, 5.64]	0.57	47%
Mixed	4	2.52 [1.36, 4.69]	0.45	87%
Medical	2	8.58 [1.56, 47.22]	0.34	95%
Nephrotoxic drugs				
Surgical	4	1.25 [0.92, 1.71]	0.34	0%
Mixed	2	2.12 [1.04, 4.32]	0.41	64%
Severity of disease*				
Surgical	5	5.69 [3.25, 8.14]	0.40	88%
Mixed	3	10.66 [3.37, 17.95]	0.35	96%
Medical	2	5.32 [3.98, 6.65]	0.18	0%
Hypotension/shock				
Surgical	4	2.48 [1.3, 4.72]	0.74	57%
Mixed	4	3.44 [1.09, 10.89]	0.79	92%
Pressors/inotropes				
Surgical	5	5.36 [2.39, 12.03]	0.81	91%
Mixed	2	3.41 [1.87, 6.20]	0.45	19%
High risk surgery/emergency surgery				
Surgical	6	3.79 [2.91, 4.94]	0.04	33%
Mixed	4	1.14 [0.64, 2.02]	0.04	91%

CI: confidence interval, SIRS: systemic inflammatory response syndrome.

**Table 5:** Sensitivity analysis.

Risk factor	Number of studies excluded	Odds ratio or mean difference* (95% CI)	*P* value for interaction	Inconsistency *I* ^2^
Age	9	4.95 [3.79, 6.12]	0.69	80%
Diabetes	4	1.58 [1.36, 1.84]	0.74	36%
Hypertension	4	1.43 [1.08, 1.89]	0.39	73%
Baseline creatinine*	3	0.14 [0.01, 0.27]	0.50	94%
Heart failure	2	2.05 [1.77, 2.38]	0.73	27%
Sepsis/SIRS	1	4.15 [2.36, 7.32]	0.72	83%
Nephrotoxic drugs	1	1.53 [1.09, 2.14]	0.97	52%
Severity of disease*	4	9.08 [4.57, 13.60]	0.43	94%
Hypotension/shock	1	3.33 [1.70, 6.52]	0.76	84%
Pressors/inotropes	2	4.52 [2.03, 10.05]	0.92	92%
High risk surgery/emergency surgery	2	2.34 [1.23, 4.49]	0.99	92%
Cardiopulmonary bypass time*	3	30.46 [23.41, 37.51]	0.74	59%
IABP	3	3.76 [2.54, 5.57]	0.64	78%

CI: confidence interval, SIRS: Systemic inflammatory response syndrome, IABP: intra-aortic balloon pump.

## List of Abbreviations

AKI: Acute kidney injury
APACHE: Acute physiologic and chronic health evaluation
BUN: Blood urea nitrogen
CC: Case control study
CI: Confidence interval
ES: Effect size
GFR: Glomerular filtration rate
EuroSCORE: European System for Cardiac Operative Risk Evaluation
HC: Historical cohort study
IABP: Intra-aortic balloon pump
ICU: Intensive care unit
IQR: Interquantile range
ISS: Injury severity score
N/A: Not applicable
NCC: Nested case control study
NOS: Newcastle-Ottawa scale
NR: Not reported
OR: Odds ratio
PC: Prospective cohort study
PRISMA: Preferred Reporting Items for Systematic Reviews and Meta-Analyses
RIFLE: Risk injury failure loss end stage kidney disease criteria
RRT: Renal replacement therapy
SD: Standard deviation
SIRS: Systemic inflammatory response syndrome.
