# Supplementary material for: Risk Factors for Development of Acute Kidney Injury in Critically Ill Patients: A Systematic Review and Meta-Analysis of Observational Studies
Source: Crit Care Res Pract. 2012 Nov 26;2012:691013. doi: 10.1155/2012/691013 (PMC3513719; doi:10.1155/2012/691013)

**APPENDIX # 1: Search**

**Ovid MEDLINE(R)** 1950 to January Week 3 2010

| **#** | **Searches** | **Results** |
| --- | --- | --- |
| 1 | Kidney Failure, Acute/ or Renal Insufficiency, Acute/ | 25863 |
| 2 | Kidney Failure, Acute/co, et, ep, pa, pp, ci, mo or Renal Insufficiency, Acute/co, et, ep, pa, pp, ci, mo | 18722 |
| 3 | Critical Illness/ | 10449 |
| 4 | exp intensive care units/ | 41255 |
| 5 | exp Critical Care/ | 34822 |
| 6 | (icu or "intensive care").mp. [mp=title, original title, abstract, name of substance word, subject heading word, unique identifier] | 83881 |
| 7 | (*Kidney Failure, Acute/ or *Renal Insufficiency, Acute/) and (6 or *Critical Illness/ or exp *intensive care units/ or exp *Critical Care/) | 1274 |
| 8 | 7 and (risk* or incidence or prevalence or mortality or probability or predict* or predispos* or rifle or afin or likelihood).mp. [mp=title, original title, abstract, name of substance word, subject heading word, unique identifier] | 705 |
| 9 | 7 and (sepsis/ or shock, septic/ or postoperative*.mp.) [mp=title, original title, abstract, name of substance word, subject heading word, unique identifier] | 224 |
| 10 | 7 and (cohort* or observation* or retrospective* or prospective*).mp. [mp=title, original title, abstract, name of substance word, subject heading word, unique identifier] | 512 |
| 11 | limit 7 to (clinical trial, all or clinical trial, phase i or clinical trial, phase ii or clinical trial, phase iii or clinical trial, phase iv or clinical trial or comparative study or meta analysis or multicenter study or practice guideline or randomized controlled trial or validation studies) | 301 |
| 12 | 8 or 9 or 10 or 11 | 934 |
| 13 | limit 12 to humans | 927 |
| 14 | (*Kidney Failure, Acute/co, et, ep, pa, pp, ci, mo or *Renal Insufficiency, Acute/co, et, ep, pa, pp, ci, mo) and 13 | 351 |

**EMBASE** 1988 to 2010 Week 03

| **#** | **Searches** | **Results** |
| --- | --- | --- |
| 1 | exp kidney injury/co, ep, et [Complication, Epidemiology, Etiology] | 2650 |
| 2 | exp acute kidney failure/co, ep, et, si [Complication, Epidemiology, Etiology, Side Effect] | 7513 |
| 3 | exp kidney injury/si or 2 or 1 | 10712 |
| 4 | exp intensive care unit/ | 32504 |
| 5 | exp critical illness/ or exp critically ill patient/ or exp intensive care/ | 207083 |
| 6 | 4 or 5 or icu*1.mp. [mp=title, abstract, subject headings, heading word, drug trade name, original title, device manufacturer, drug manufacturer name] | 227409 |
| 7 | 3 and 6 | 1286 |
| 8 | 7 and ((risk* or incidence).mp. or prevalence/ or mortality.mp. or probability.mp. or likelihood.mp. or predict*.mp. or redispos*.mp. or rifle.mp. or afin.mp.) [mp=title, abstract, subject headings, heading word, drug trade name, original title, device manufacturer, drug manufacturer name] | 800 |
| 9 | 7 and (sepsis.mp. or septic shock/ or postoperative*.mp.) [mp=title, abstract, subject headings, heading word, drug trade name, original title, device manufacturer, drug manufacturer name] | 529 |
| 10 | 8 or 9 | 968 |
| 11 | 10 and (case-series/ or systematic review/ or meta-analysis/ or cohort*.mp. or observation*.mp. or retrospective*.mp. or prospective*.mp.) [mp=title, abstract, subject headings, heading word, drug trade name, original title, device manufacturer, drug manufacturer name] | 332 |
| 12 | 10 and (major clinical study/ or comparative study/ or exp clinical trial/ or multicenter study.mp.) [mp=title, abstract, subject headings, heading word, drug trade name, original title, device manufacturer, drug manufacturer name] | 481 |
| 13 | 11 or 12 | 554 |
| 14 | ..l/ 13 hu=y | 554 |
| 15 | (exp *kidney injury/si or exp *acute kidney failure/co, ep, et, si or exp *kidney injury/co, ep, et) and 14 | 244 |
| 16 | 14 and (*kidney injury/ or *acute kidney failure/) | 244 |
| 17 | 15 or 16 | 244 |

**EBM Reviews - Cochrane Central Register of Controlled Trials** 4th Quarter 2009

| **#** | **Searches** | **Results** |
| --- | --- | --- |
| 1 | kidney failure, acute/ or (acute adj2 (renal or kidney)).mp. [mp=title, original title, abstract, mesh headings, heading words, keyword] | 1170 |
| 2 | (icu or icus).mp. or critical illness/ or (criticially adj ill*).mp. or (critical adj care).mp. [mp=title, original title, abstract, mesh headings, heading words, keyword] | 2360 |
| 3 | 1 and 2 | 100 |
| 4 | 3 and (risk* or complicat* or predispos* or hospitali* or afin or rifle or postoperative* or sepsis or septic or predict*).mp. [mp=title, original title, abstract, mesh headings, heading words, keyword] | 39 |

**SCOPUS**

Your query: (TITLE-ABS-KEY(("acute renal" OR "acute kidney") AND (complicat* OR risk* OR likelihood OR etiology OR rifle OR afin OR hospitaliz* OR hospitalis* OR septic OR sepsis OR postoperative*)) AND TITLE-ABS-KEY("critically ill" OR "critical illness" OR icu OR icus OR "intensive care" OR "critical care") AND TITLE-ABS-KEY((outcome* OR mortality OR predict* OR probability OR incidence OR predispos*) AND (observation* OR "case series" OR cohort* OR prospective* OR retrospective*))) AND TITLE("critically ill" OR "critical illness" OR icu OR icus OR "intensive care" OR "critical care" OR icu OR intensive OR critical* OR "acute kidney" OR "acute renal")

**APPENDIX #2 Funnel Plots**

1-Funnel plot for age:


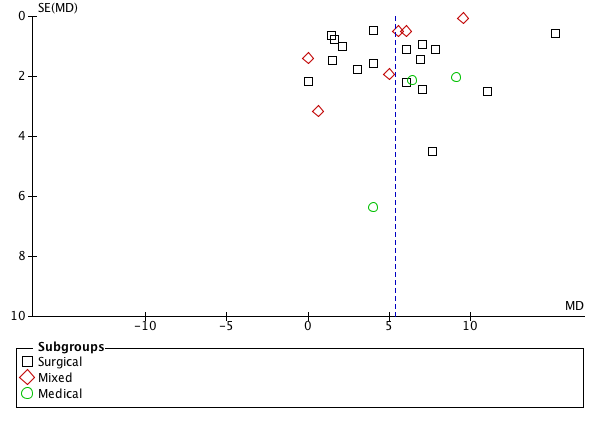


2-Funnel plot for diabetes:


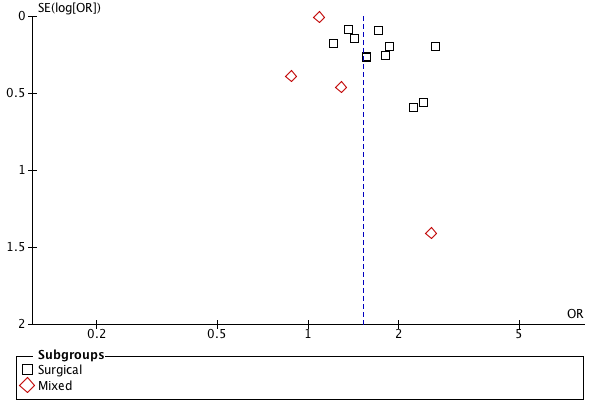


3- Funnel plot for hypertension:


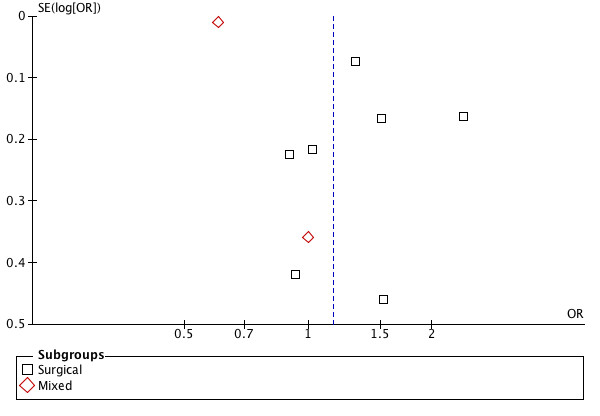


4- Funnel plot for baseline creatinine:


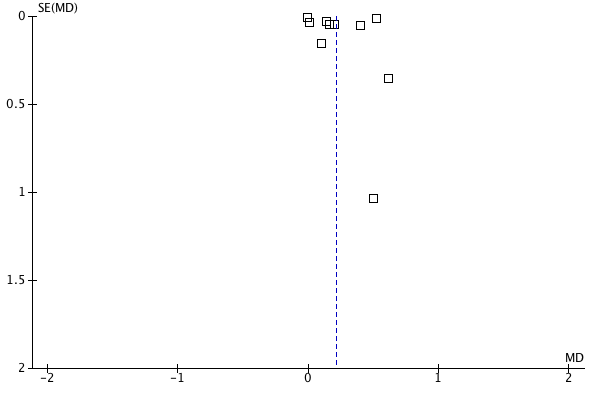


5- Funnel plot for heart failure:


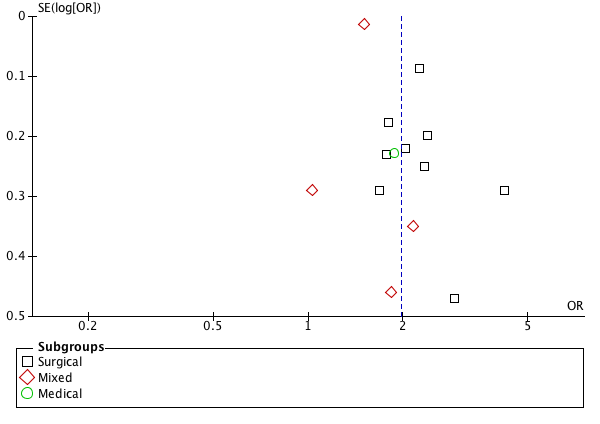


6- Funnel plot for Sepsis/SIRS:


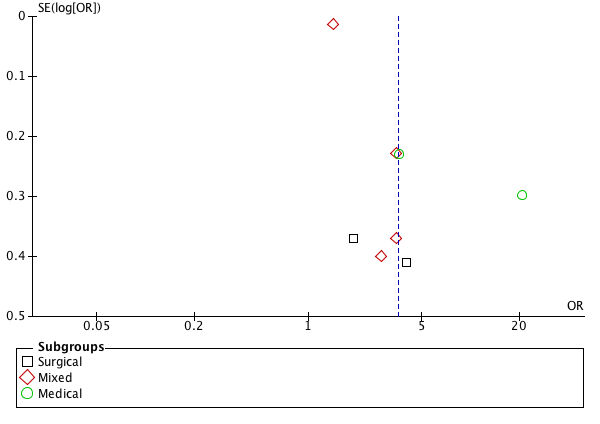


7- Funnel plot for nephrotoxic drugs:


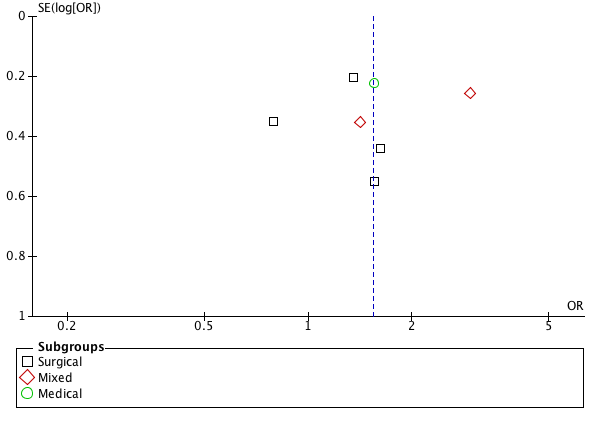


8- Funnel plot for severity of disease:


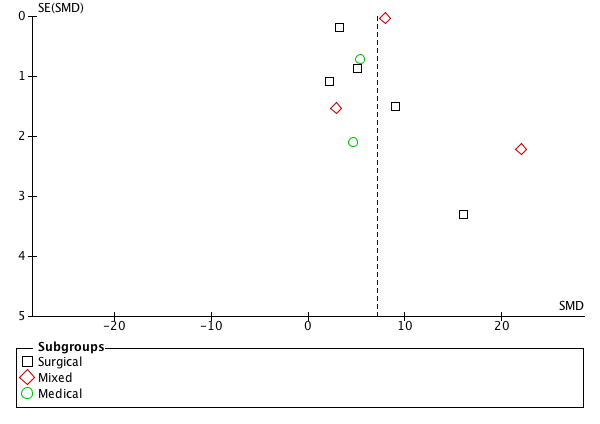


9- Funnel plot for hypotension/shock:


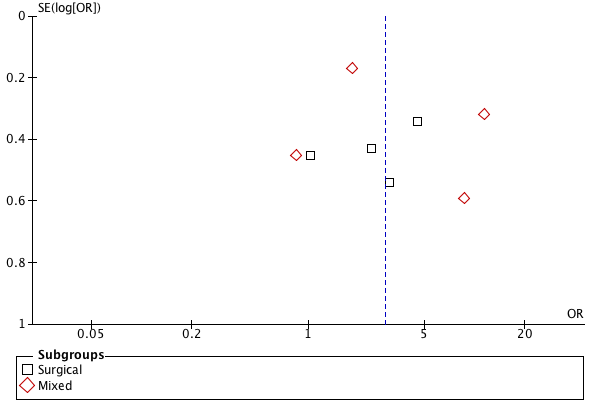


10- Funnel plot for pressors/inotropes:


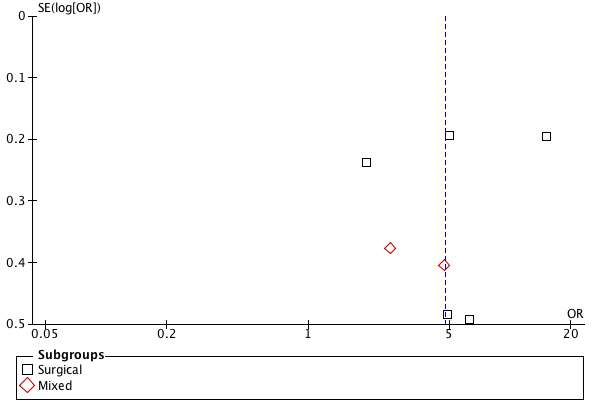


11- Funnel plot for high-risk surgery/emergency surgery:


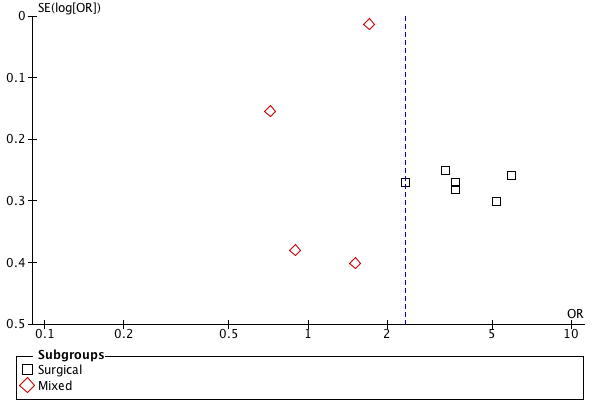


12- Funnel plot for cardiopulmonary bypass time:


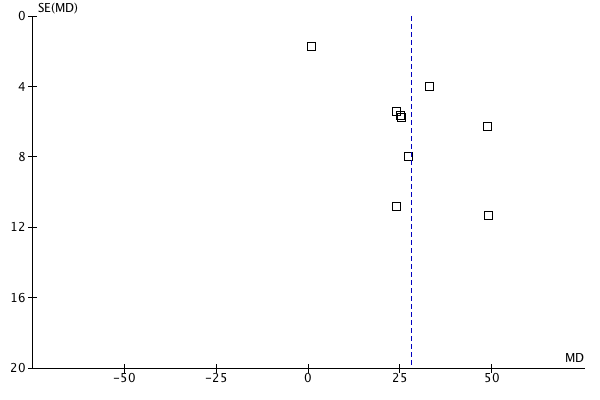


13- Funnel plot for IABP:


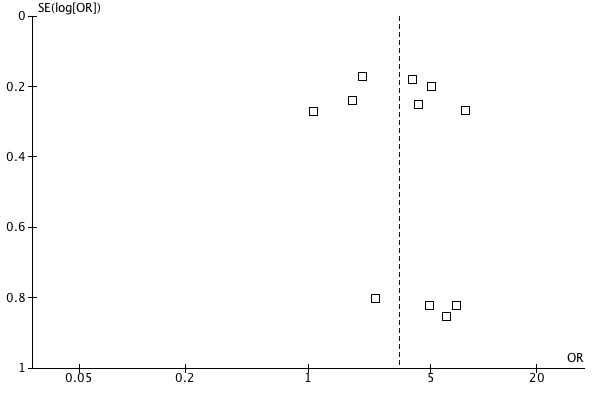

Supplement: Supplementary file 1 — Appendix 1 includes the detailed keywords and search strategy employed by the expert librarian in the following databases: Ovid MEDLINE, Ovid EMBASE, Cochrane Library, Web of Science, and Scopus. Appendix 2 includes the funnel plots employed for critically appraising the publication bias for each identified risk factor for acute kidney injury with the respective subgroups, which include the ICU setting: surgical, medical or mixed. [file 691013.f1.doc]
